# Effects of Microencapsulated Ferulic Acid or Its Prodrug Methyl Ferulate on Neuroinflammation Induced by Muramyl Dipeptide

**DOI:** 10.3390/ijerph191710609

**Published:** 2022-08-25

**Authors:** Giada Botti, Anna Bianchi, Barbara Pavan, Paola Tedeschi, Valentina Albanese, Luca Ferraro, Federico Spizzo, Lucia Del Bianco, Alessandro Dalpiaz

**Affiliations:** 1 Department of Chemical, Pharmaceutical and Agricultural Sciences, University of Ferrara, via Fossato di Mortara 19, I-44121 Ferrara, Italy; 2 Department of Neuroscience and Rehabilitation—Section of Physiology, University of Ferrara, via L. Borsari 46, I-44121 Ferrara, Italy; 3 Department of Environmental and Prevention Sciences, University of Ferrara, Corso Ercole I d’Este 32, I-44121 Ferrara, Italy; 4 Department of Life Sciences and Biotechnology, University of Ferrara and LTTA Center, via Fossato di Mortara 19, I-44121 Ferrara, Italy; 5 Department of Physics and Earth Science, University of Ferrara, via G. Saragat 1, I-44122 Ferrara, Italy

**Keywords:** ferulic acid, methyl ferulate, prodrug, antioxidant, solid lipid microparticles, inflammation, TNF-α

## Abstract

Ferulic acid (Fer) is known for its antioxidant and anti-inflammatory activities, which are possibly useful against neurodegenerative diseases. Despite the ability of Fer to permeate the brain, its fast elimination from the body does not allow its therapeutic use to be optimized. The present study proposes the preparation and characterization of tristearin- or stearic acid-based solid lipid microparticles (SLMs) as sustained delivery and targeting systems for Fer. The microparticles were produced by conventional hot emulsion techniques. The synthesis of the methyl ester of Fer (Fer-Me) allowed its encapsulation in the SLMs to increase. Fer-Me was hydrolyzed to Fer in rat whole blood and liver homogenate, evidencing its prodrug behavior. Furthermore, Fer-Me displayed antioxidant and anti-inflammatory properties. The amount of encapsulated Fer-Me was 0.719 ± 0.005% or 1.507 ± 0.014% in tristearin or stearic acid SLMs, respectively. The tristearin SLMs were able to control the prodrug release, while the stearic acid SLMs induced a significant increase of its dissolution rate in water. Jointly, the present results suggest that the tristearin SLMs loaded with Fer-Me could be a potential formulation against peripheral neuropathic pain; conversely, the stearic acid SLMs could be useful for Fer-Me uptake in the brain after nasal administration of the formulation.

## 1. Introduction

Inflammation is defined as a physiological response of the body to tissue injury, pathogen invasion and irritants. During inflammation, immune cells of the innate and/or adaptive immune system are activated and recruited to the site of inflammation, which exhibits an important contribution to a wide range of diseases, such as central and peripheral neurodegenerative diseases [1]. Autoinflammatory disorders are a new and expanding class of inflammatory diseases characterized by recurrent episodes of systemic inflammation in the absence of pathogens, autoantibodies, or antigen specific T cells. These disorders are caused by primary dysfunction of the innate immune system, without evidence of adaptive immune dysregulation. Innate immune abnormalities include the following: (i) aberrant responses to pathogen-associated molecular patterns (PAMPs), such as lipopolysaccharide (LPS) or the decomposition product released during the growth and division of Gram-negative and Gram-positive bacterial peptidoglycan (the muramyl dipeptide, MDP), and (ii) dysregulation of inflammatory cytokines, such as interleukin (IL)-1β and tumor necrosis factor alpha (TNF-α), or their receptors [2]. It is becoming clear that all neurodegenerative diseases have a dominant inflammatory phenotype involved in immune-based mechanisms of Alzheimer’s disease (AD), amyotrophic lateral sclerosis (ALS), and Parkinson’s disease (PD). It is particularly striking that recent mechanistic studies into these debilitating diseases have provided common nodes of innate immune cell dysfunction occurring in autoinflammatory disorders, yielding important insights into immune modulation therapeutic strategies [3]. Notably, increasing evidence suggests that neuroinflammation is also tightly linked to oxidative stress, being induced by the excessive accumulation of reactive oxygen species (ROS) and mediated by the transcription factor NF-κB signaling, along with the production of a series of pro-inflammatory cytokines, such as IL-1β, IL-6, and TNF-α [4]. TNF-α, in particular, is a pluripotent cytokine that is an early responder of the innate immune system and stimulates the expression of other pro-inflammatory cytokines. In the healthy brain, TNF-α expression is low, but it is elevated in multiple disease states, such as AD [3]. TNF-α production is also induced by MDP, by entering the cells and resulting in inflammatory damage via nucleotide-binding oligomerization domain 2 (intracellular receptor NOD2), a member of NOD-like receptors (NLRs) [5]. Once activated by MDP, NOD2 triggers immune responses through the activation of NF-kB and mitogen-activated protein kinases (MAPKs) signaling cascades. It is indeed known that NF-κB signaling is up-regulated by MDP [6] and down-regulated by natural polyphenols, such as ferulic acid (Fer) [7]. MDP was also found in rheumatoid arthritis patients’ synovial tissue, which contributes to the pathogenesis of this disease through NOD2 signaling activation, together with the development of neuropathic pain by producing pronociceptive cytokines, such as TNF-α [5]. Given the fundamental role of neuroinflammation in the development of neuropathic pain, there is growing interest in targeting the neuroimmune interface for pain management [5].

Fer belongs to the class of phenolic acids, and it is known for its low toxicity, joined to anti-inflammatory (via down regulation of NF-κB signaling) and antioxidant activities [8,9]. About the antioxidant activity, Fer shows an important role as a scavenger of ROS, enhancer of physiological scavenger enzymes, or inhibitor of processes involved in the production of ROS [8,10,11]. Fer is, therefore, proposed for the prevention and therapy of both cardiovascular and neurodegenerative diseases [12,13,14], that could both benefit from its antioxidant and anti-inflammatory properties.

The high absorption rate and potentially high oral bioavailability [13,14,15] of Fer, together with its known ability to permeate in the central nervous system (CNS) [13,16,17], suggest the promising potential therapeutic activity of this drug, both at peripheral and central levels. On the other hand, Fer is characterized by a fast elimination rate from the body, showing half-life values of approximatively 30 min in both humans and rodents [12,14,17]. The permanence of Fer at plasma level and in the CNS appears, therefore, low and poorly adequate for therapeutic proposals [17,18], despite the ability of this compound to be rapidly absorbed in the oral way and to cross the blood brain barrier.

Several strategies have been proposed to overcome the poor permanence of Fer in the body. As an example, self-assembled nanoparticles composed of Fer modified glycol chitosan were studied for functional restoration of injured spinal cord [19]. A self-microemulsifying drug delivery system was evaluated as a carrier to improve the pharmacokinetic behavior and anti-insomnia efficacy of Fer in [17]. Nanostructured lipid carriers loaded with Fer were evaluated for their therapeutic potential against ischemic stroke [20] and chitosan-coated solid lipid nanoparticles loaded with Fer were investigated for the management of AD [21]. Alternatively, a prodrug approach was proposed to deliver Fer into the mouse brain via the L-type amino acid transporter (LAT1) [22].

The present study focuses on solid lipid microparticles (SLMs) as an alternative carrier system for Fer. These micrometer-sized particles consist of a solid fat core, based on biocompatible and biodegradable natural lipids, stabilized by a layer of surfactant molecules on the surface [23]. This type of carrier is characterized by very simple formulation and purification methods, easily reproducible by the pharmaceutical industry, allowing sustainable practices for the environment. Due to their hydrophobic nature, SLMs attain poor entrapment capacity for hydrophilic drugs [23,24,25], whereas lipophilic compounds are greatly incorporated in the lipid particles [23,26].

The difficulties related to the incorporation of hydrophilic drugs in SLMs can be overcome with the synthesis of lipophilic prodrugs [27,28,29]. For this reason, the methyl ester derivative as a potential prodrug of Fer (methyl ferulate, Fer-Me) was synthesized, in order to evaluate its aptitude to increase Fer loading in SLMs. Fer-Me was therefore characterized by evaluating its potential prodrug behavior in physiologic fluids, such as rat whole blood or liver and brain homogenates. Moreover, the potential antioxidant activity of Fer-Me was investigated and its anti-inflammatory power was analyzed by using an in vitro model of neuroinflammation, based on MDP stimulation of PC12 cells, chosen as a model for neural differentiation, and related production of TNF-α. SLMs based on tristearin or stearic acid were loaded with Fer or Fer-Me and characterized by scanning electron microscopy (SEM), powder X-ray diffraction analysis, and differential scanning calorimetry (DSC). The ability of the SLMs to modulate the dissolution or release rate of Fer or Fer-Me was investigated. The results of these studies allowed the proposition of appropriate methods of administration of SLMs to target the therapeutic effects of Fer, or its derivatives, in specific sites of the body.

## 2. Materials and Methods

### 2.1. Materials

Ferulic and caffeic acids, acetic acid, dimethyl sulfoxide (DMSO), trifluoroacetic acid (TFA) and Trizma Base were obtained from Sigma-Aldrich (Milan, Italy). The chemicals were purchased from BLD Pharmatech GmbH (Kaiserslautern, Germany) or Sigma-Aldrich (Milan, Italy). DPPH (2,2-diphenyl-1-picrylhydrazyl) and Trolox (6-hydroxy-2,5,7,8-tetramethylchroman-2-carboxylic acid) were obtained from Merck Life Science (Milan, Italy). Methanol, acetonitrile (CH_3_CN), ethyl acetate (EtOAc) and water were of high-performance liquid chromatography (HPLC) grade from Carlo Erba Reagents S.A.S. (CEDEX, France). Male Wistar rats were purchased from Charles River laboratories (Calco, Italy). Tristearin, stearic acid and Tween 60 were supplied by Merck (Damstad, Germany). All other reagents and solvents were of analytical grade (Sigma-Aldrich). Media, sera, reagents, and vessels for cell culture were obtained from Microtech (Naples, Italy) and Thermo Fisher Scientific (Milan, Italy).

### 2.2. General Procedure for the Synthesis of Methyl Caffeate (***3***) and Methyl Ferulate (***4***) via Fischer Esterification

Reaction mixtures were monitored by thin-layer chromatography (TLC) on silica gel (precoated F254 Macherey-Nagel plates) and visualized with a UV lamp (254 nm light source). Compounds were purified through silica gel flash chromatography (silica gel 60, 40−63 μm) using opportune eluent mixtures. Mass spectra were recorded on a Waters ESI Micromass ZQ, dissolving the samples in a solution of H_2_O/CH_3_CN/TFA (40:60:0.1). NMR analyses were performed on a Varian 400 MHz spectrometer in DMSO-d_6_. Chemical shifts (δ) are reported in parts per million (ppm) using the peak of tetramethylsilane as an internal standard in deuterated DMSO, and coupling constants (J) are reported in Hertz (Hz). Splitting patterns are designed as: s, singlet; d, doublet; m, multiplet.

**Figure 1:**
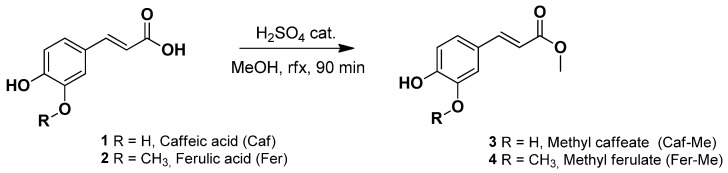
Synthesis of Caf-Me (**3**) and Fer-Me (**4**) via Fischer esterification.

A catalytic amount (0.5 mL) of concentrated sulfuric acid (95–98%) was added at 0 °C to a stirring solution of caffeic acid (**1**) or ferulic acid (**2**) (5 mmol) in methanol (11 mL) (Figure 1). The reaction was heated at 80 °C (reflux) for 90 min and then cooled to room temperature. The solvent was removed under vacuum giving a brownish powder for **1** and a yellow oil for **2**. The crude was dissolved in EtOAc (20 mL), and the resulting organic phase was washed with water (3 × 20 mL), aqueous sodium bicarbonate 5% *w*/*v* (3 × 20 mL), dried over anhydrous magnesium sulphate, and concentrated to dryness. The crude was purified via flash column chromatography on a silica gel using the appropriate mixture of EtOAc and Petroleum ether as eluent (see Supplementary Materials).

### 2.3. Antioxidant Activity

The antioxidant activity was measured by the DPPH assay, according to the method of Fukumoto and Mazza [30], with minor modifications. A deep purple solution of DPPH 2,2-diphenyl-1-picrylhydrazyl (0.06 mM) was prepared in methanol and the absorbance was measured at 515 nm (Jasco V630PC spectrophotometer, Tokyo, Japan) as a control. Trolox (6-hydroxy-2,5,7,8-tetramethylchroman-2-carboxylic acid, a water-soluble analog of vitamin E) was used as a reference compound and its solutions in methanol (0.05–1 mM) were used to prepare a calibration curve. Aliquots (50 µL) of the increasingly concentrated solutions of standard were added to 1450 µL of DPPH methanol solution; the mixture was stirred vigorously and kept for 15 min in the dark at room temperature. The decrement of spectrophotometric absorbance with the color decrease toward yellow was then registered. The samples of Fer and Fer-Me (1 mM in methanol solution) were treated in the same way. The antioxidative activity was calculated as a percentage of inhibition of the DPPH radical and Trolox mM equivalent antioxidant capacity, according to the following Equation (1):

(1) $$\%\;\mathrm{inhibition}=\frac{A_{t=0\min}-A_{t=15\;\min}}{A_{t=0\;\min}}\;\cdot 100$$

where A_t=0 min_ was the absorbance of the control (DPPH) and A_t=15 min_ was the absorbance of the standard or the sample. All the experiments were performed in triplicate.

### 2.4. HPLC Analysis

The quantification of Fer-Me and Fer was performed by HPLC. The chromatographic apparatus consisted of a modular system (model LC-10 AD VD pump and model SPD-10A VP variable wavelength UV−vis detector; Shimadzu, Kyoto, Japan) and an injection valve with a 20 μL sample loop (model 7725; Rheodyne, IDEX, Torrance, CA, USA). Separations were performed at room temperature on a 5 μm Force Biphenyl column (150 mm × 4.6 mm i.d.; Restek, Milan, Italy), equipped with a guard column packed with the same Force Biphenyl material. Data acquisition and processing were accomplished with a personal computer using CLASS-VP Software, version 7.2.1 (Shimadzu Italia, Milan, Italy). The detector was set at 320 nm. The mobile phase consisted of a mixture of 0.4% acetic acid in water and methanol regulated by a gradient profile, programmed as follows: isocratic elution with 60% (*v*/*v*) MeOH in acid H_2_O for 5 min; then, a 5 min linear gradient to 80% (*v*/*v*) MeOH in acid H_2_O, immediately followed by a 5 min linear gradient to 60% (*v*/*v*) MeOH in acid H_2_O. After each cycle the column was conditioned with 60% (*v*/*v*) MeOH in acid H_2_O for 10 min. The flow rate was 0.8 mL/min. Caf-Me was used as internal standard for the analysis of rat blood and liver or brain homogenate extracts (see below). The retention times for Fer, Caf-Me and Fer-Me were 4.1, 5.1, and 9.1 min, respectively. The evaluation of chromatographic precision and the calibration method are described in the Supplementary Materials.

### 2.5. Ferulic Acid and Methyl Ferulate Stock Solutions

Stock solutions of 50 mM of Fer and Fer-Me in DMSO were prepared and stored at −20 °C until their use for kinetic studies.

### 2.6. Kinetic Analysis in Tris-HCl

Fer or Fer-Me were incubated at 37 °C in 30 mL of Tris-HCl buffer (pH 7.4), contained in centrifuge conical tubes with screw cups. The concentration of incubation of the compounds was 100 μM, obtained by adding to the buffer solution the appropriate amounts of stock solutions in DMSO. The samples were stirred mechanically (100 rpm). At regular time intervals, 200 μL of samples were withdrawn and, after filtration (regenerate cellulose, 0.45 μm), 10 μL aliquots were immediately injected into the HPLC apparatus for the quantification of Fer or Fer-Me. All the values were obtained as the mean of three independent experiments.

### 2.7. Kinetic Analysis in Whole Blood

Fer-Me or Fer were incubated at 37 °C in heparinized whole blood obtained from different male Wistar rats weighing 200−250 g. Three milliliters of whole blood were spiked with compound solutions, resulting in a final concentration of 30 μM, obtained by adding the appropriate amounts of 50 mM stock solution in DMSO. At regular time intervals, 100 μL of samples were withdrawn and immediately quenched in 500 μL of ice-cold water, and, then, 50 μL of 10% sulfosalicylic acid and 50 μL of internal standard (100 μM Caf-Me dissolved in a mixture of MeOH and H_2_O 50:50 *v*/*v*) were added. The samples were extracted twice with 1 mL of water-saturated ethyl acetate, and, after centrifugation (13,500× *g* for 10 min), the organic layer was reduced to dryness under a nitrogen stream. Two hundred microliters of a water and methanol mixture (50:50 *v*/*v*) were added, and, after centrifugation (16,000× *g* for 5 min), 20 μL was injected into the HPLC system. All the values were obtained as the mean of three independent incubation experiments. The accuracy and calibration of the analytical method are described in the Supplementary Materials.

### 2.8. Preparation of Rat Liver Homogenates

Male Wistar rats were sacrificed by decapitation and their livers were immediately isolated, washed with ice-cold saline solution and homogenized in 4 volumes (*w*/*v*) of Tris HCl (50 mM, pH 7.4, 4 °C) by using a Potter-Elvehjem apparatus. The supernatant obtained after centrifugation (2000× *g* for 10 min at 4 °C) was decanted off and stored at −80 °C before its use for kinetic studies. The total protein concentration in the tissue homogenate was determined using the Lowry procedure [31] and resulted as 31.2 ± 1.2 μg protein/μL.

### 2.9. Preparation of Rat Brain Homogenates

Male Wistar rats were sacrificed by decapitation and their brains were immediately isolated and homogenized in 5 volumes (*w*/*v*) of Tris HCl (50 mM, pH 7.4, 4 °C) with an ultra-Turrax (IKA Werke GmbH & Co. KG, Staufen, Germany) using 3 × 15 s bursts. The supernatant obtained after centrifugation (3000× *g* for 15 min at 4 °C) was decanted off and stored at −80 °C before its use for kinetic studies. The total protein concentration in the tissue homogenate was determined using the Lowry procedure [31] and resulted as 7.1 ± 0.3 μg protein/μL.

### 2.10. Kinetic Analysis in Rat Brain or Rat Liver Homogenates

Fer-Me or Fer were incubated at 37 °C in 3 mL of rat brain or rat liver homogenates, resulting in a final concentration of 30 μM, obtained by adding the appropriate amounts of stock solution in DMSO. At regular time intervals, 100 μL of samples were withdrawn and immediately quenched in 250 μL of ice-cold water and, then, 50 μL of 10% sulfosalicylic acid and 50 μL of internal standard (100 μM Caf-Me dissolved in a mixture of MeOH and H_2_O 50:50 *v*/*v*) were added. The samples were extracted twice with 1 mL of water-saturated ethyl acetate, and, after centrifugation (13,500× *g* for 10 min), the organic layer was reduced to dryness under a nitrogen stream. Two hundred microliters of a water and methanol mixture (50:50 *v*/*v*) was added, and, after centrifugation (16,000× *g* for 5 min), 20 μL was injected into the HPLC system. All the values were obtained as the mean of three independent incubation experiments. The accuracy and calibration of the analytical method are described in the Supplementary Materials.

### 2.11. Kinetic Calculations

The half-lives of Fer-Me incubated in the rat whole blood or liver homogenates were calculated by nonlinear regression (exponential decay) of residual percentage values over time and confirmed by linear regression of the log concentration values versus time. The quality of the fits was determined by evaluating the correlation coefficients (r) and *p* values. All the calculations were performed by using the computer program GraphPad Prism 7 (GraphPad, San Diego, CA, USA).

### 2.12. PC12 Cell Culture and Treatment

PC12 cell line (RRID:CVCL_0481), derived from rat adrenal gland pheochromocytoma, was a kind gift of Dr Federica Brugnoli from the Department of Translational Medicine, University of Ferrara, Ferrara (Italy). The cells were grown in RPMI-1640 (HiGlutaXL) medium (Microtech, Naples, Italy), supplemented with 10% horse serum (HS), and 5% fetal bovine serum (FBS). PC12 cells were cultured by detaching them from the collagen IV-coated flask with a rubber scraper, transferring the cell suspension to a Falcon tube and pipetting them through a sterile tip to avoid any clumping, then splitting and seeding them into separate flasks. To differentiate into neuronal phenotypes, PC12 cells were seeded in 24-well collagen IV-coated culture plates at 5 × 10^3^ cells/well density, and were then left to adhere to the surface of the well for 24 h. The next day they were washed once with serum free Dulbecco’s Modified Eagle’s Medium (DMEM) medium and then switched to DMEM medium, containing 100 ng/mL nerve growth factor (NGF) and 1% HS up to 7 days of differentiation period. All the cultures were maintained at 37 °C in a humidified 5% CO_2_ atmosphere.

About the inflammation assay, 5 µg/mL muramyl dipeptide (DPI, Merck, Milan, Italy) was applied to induce inflammatory injury in 7 day differentiated PC12 cells, in the absence, and in the presence, of increasing concentrations of Fer-Me (10, 25, 50 µM) for 24 h.

### 2.13. Enzyme-Linked Immunosorbent Assay (ELISA)

To remove cell debris, the samples of 1 mL incubation medium at the end of treatments were immediately centrifuged for 20 min at 1000× *g* at 4 °C. The cell-free culture supernatants were collected and stored at −20 °C until concentrations of inflammatory TNF-α present in the incubation media were detected by means of an ELISA assay, based on the sandwich ELISA principle with Avidin-Horseradish Peroxidase (HRP) conjugate detection of biotinylated antibody specific for Human TNF-α, following manufacturer’s instructions (Elabscience Biotechnology Inc.; catalog no: E-EL-H0109; purchased from Microtech Srl, Naples, Italy) at 450 nm using a microtiter plate reader (Sunrise^®^ Microplate Reader, Tecan Trading AG, Männedorf, Switzerland). Results are presented as mean ± S.E.M. values of four independent experiments. Data were plotted and analyzed with GraphPad Prism 7 (GraphPad, San Diego, CA, USA). Statistical analysis was performed by one-way analysis of variance (ANOVA), followed by Bonferroni’s multiple comparisons test. Significance was set at *p* < 0.05.

### 2.14. Preparation of Ferulic Acid or Methyl Ferulate Loaded Microparticles

SLMs were prepared by adding hot (75−85 °C) deionized water (18.75 mL), containing 0.7% (*w*/*w*) Tween 60 to the melted lipid phase (1.125 g of tristearin or stearic acid) in which Fer or Fer-Me (30 mg) had been dispersed. The sample was subjected to high-shear mixing (21,500 rpm for 2 min) with an Ultra-Turrax T25 (IKA-Werk, Staufen, Germany) at 75−85 °C, and the obtained emulsion was rapidly cooled at room temperature, under magnetic stirring. The formed suspension was centrifuged (10,000× *g* for 15 min) to recover the SLMs, which were freeze-dried to give water-free microparticles. Unloaded particles were also prepared with the same procedure without the drugs.

### 2.15. Microparticle Characterization

The morphology of the microspheres was determined by observation on a scanning electron microscope equipped with a lanthanum hexaboride (LaB_6_) emitter (HV-SEM; Zeiss EVO40XVP, Arese, Milan, Italy). The samples were placed on double-sided tape that had previously been secured to aluminum stubs and then analyzed at 20 kV acceleration voltage after gold sputtering.

### 2.16. Ferulic Acid or Methyl Ferulate Content in the SLMs

The content of Fer or Fer-Me in the microparticulate powders was determined by the following method [28]. The microparticles (about 5 mg) were accurately weighed using a high precision analytical balance (d = 0.01 mg; Sartorius, model CP 225D, Goettingen, Germany), and dissolved in methanol at 80 °C for 15 min. The samples were then cooled at room temperature, and the final volume of the solution was adjusted at 2 mL. Then, 10 μL of filtered solutions (regenerate cellulose, 0.45 μm) was injected into the HPLC system for Fer or Fer-Me quantification. The drug loading and entrapment efficiency were calculated according to the following Equations (2) and (3):

(2) $$\mathrm{Drug}\;\mathrm{loading}\;\left(\frac{W}{W}\right)(\%)=\frac{\mathrm{mass}\;\mathrm{of}\;\mathrm{drug}\;\mathrm{in}\;\mathrm{microparticles}}{\mathrm{mass}\;\mathrm{of}\;\mathrm{loaded}\;\mathrm{microparticles}}\times 100$$

(3) $$\mathrm{Entrapment}\;\mathrm{efficiency}\;\left(\%\right)=\frac{\mathrm{mass}\;\mathrm{of}\;\mathrm{drug}\;\mathrm{in}\;\mathrm{microparticles}}{\mathrm{starting}\;\mathrm{mass}\;\mathrm{of}\;\mathrm{drug}}\times 100$$

All the values obtained are the mean of four independent experiments.

### 2.17. Powder X-ray Diffraction Analysis

Powder diffraction spectra analysis was performed for stearic acid, tristearin, Fer, Fer-Me, the mixtures of Fer with tristearin or stearic acid, the mixtures of Fer-Me with tristearin or stearic acid and the microparticles of stearic acid or tristearin loaded with Fer or Fer-Me. The amounts of the lipids (tristearin of stearic acid) and drugs (Fer or Fer-Me) were mixed with the same ratio chosen for the formulation of microparticles (Section 2.14). The spectra were recorded, at room temperature, on a Bruker D-8 Advance diffractometer with graphite monochromatized Cu Kα radiation (λ = 1.5406 Å). The data were recorded at 2θ steps of 0.02° with 1 s/step.

### 2.18. Differential Scanning Calorimetry

Differential scanning calorimetry (DSC) measurements were carried out using a Netzsch Thermal Analyzer (STA 409). The DSC signal was calibrated using an indium standard. The samples had a typical mass of 4−6 mg, measured using a high precision analytical balance (d = 0.01 mg; Sartorius, model BP 210D, Goettingen, Germany). They were put in non-hermetic aluminum pans and scanned at a heating rate of 3 °C/min in the 40−200 °C range under a continuous purged dry nitrogen flux (20 mL/min).

### 2.19. Kinetic Analysis in Phosphate Buffer Saline

Fer or Fer-Me were incubated at 37 °C in 30 mL of PBS (pH 7.4) contained in centrifuge conical tubes with screw cups. The concentration of incubation of the compounds was 100 μM, obtained by adding to PBS the appropriate amounts of stock solutions in DMSO. The samples were stirred mechanically (100 rpm). At regular time intervals 200 μL of samples were withdrawn and, after filtration (regenerate cellulose, 0.45 μm), 10 μL aliquots were immediately injected into the HPLC apparatus for the quantification of Fer or Fer-Me. All the values were obtained as the mean of three independent experiments.

### 2.20. In Vitro Dissolution and Release Studies from SLMs

An accurately weighed amount of Fer or Fer-Me (about 0.6 mg weighed with the analytical balance Sartorius CP 225D), or microparticles containing an equivalent quantity of encapsulated substances, were added to 30 mL of PBS (pH 7.4). The samples were maintained at 37 °C and stirred mechanically (100 rpm). Aliquots (200 μL) were withdrawn at fixed time intervals, and 10 μL of filtered samples (regenerate cellulose, 0.45 μm) was injected into the HPLC system. An equal volume of medium was added after each sampling to maintain sink conditions. The collected samples were quantified for Fer and Fer-Me using the developed HPLC method. All the values obtained were the mean of four independent experiments.

## 3. Results and Discussion

### 3.1. Synthesis of Methyl Ferulate and Methyl Caffeate

Considering that the drug loading of lipidic carriers can be optimized by using lipophilic prodrugs [27,28,29], the synthesis and characterization of Fer-Me as the simplest potential lipophilic prodrug of Fer is here described.

The aptitude of Fer-Me to be a prodrug of Fer was studied in physiologic fluids. In particular, its hydrolysis was quantified via HPLC; the extraction and analytical procedures related to the quantification required, as internal standard, the use of Caf-Me which was therefore synthesized.

The synthesis of Fer-Me and Caf-Me were carried out via Fischer esterification (described in Section 2.2) following a well-known procedure from literature, which gave the desired products in high yields (about 75%) [32,33]. In general, Fischer esterification is a very simple and low-cost one-pot reaction, with low environmental impact in terms of waste products and harmfulness of the reagents, compared to other methods, such as esterification via acyl-chloride. Fischer esterification requires only a catalytic amount of sulfuric acid, compared to esterification via acyl-chloride, that requires stoichiometric amounts of thionyl chloride, leading to yields similar to those obtained via Fischer esterification [34,35].

### 3.2. Antioxidant Activity

The characterization of Fer-Me included the analysis of its antioxidant activity, taking into account that lipophilic derivatives of Fer obtained by its conjugation with fatty alcohols, such as butanol, are known to decrease in vitro the production of ROS in a model of AD [36].

DPPH is a relatively stable radical that can be easily reduced to the corresponding hydrazine by abstracting a hydrogen from hydrogen donors. The DPPH assay is widely used to evaluate the antioxidant activity of phenolic molecules or their ability to transfer labile protons to radicals. The DPPH inhibition test was used to evaluate the antioxidant capacity of Fer and Fer-Me. According to the method described in Section 2.3, the % inhibition of DPPH as such, or expressed as mM of Trolox equivalent, was taken as the reference standard, and was evaluated. Fer was shown to inhibit the radical DPPH by 49.21 ± 0.79% at a concentration of 33 µM, equal to 0.507 ± 0.008 mM Trolox equivalent. At the same concentration, Fer-Me inhibited DPPH by 43.75 ± 1.5%, equal to 0.450 ± 0.015 mM Trolox equivalent. Fer-Me evidenced, therefore, an antioxidant power of about 90%, in comparison to that of Fer. These results suggested that Fer-Me might induce in vivo antioxidant effects similar to that evidenced for Fer.

### 3.3. Hydrolysis Studies of Methyl Ferulate

The following step of the work here described was the evaluation of the potential hydrolysis pattern of Fer-Me in different physiologic media, such as rat whole blood, or rat brain and liver homogenates, in order to investigate its potential prodrug behavior. In this aim, it was necessary to detect and quantify all incubation media, not only the prodrug but also its potential hydrolysis product, i.e., Fer. To this purpose, an efficacious analytical method was developed, based on the use of a reverse phase Force Biphenyl HPLC column and a mobile phase following a gradient profile. No interferences were observed from whole blood and brain or liver homogenate extract components. The chromatographic precision and calibration data obtained for Fer and Fer-Me are reported in Supplementary Materials. Fer or Fer-Me were not degraded in Tris-HCl buffer (pH 7.4), during their incubation at 37 °C for eight hours. This result meant that any potential degradation observed for Fer or Fer-Me in rat brain or liver homogenates could not be attributed to the buffer solution.

**Figure 2:**
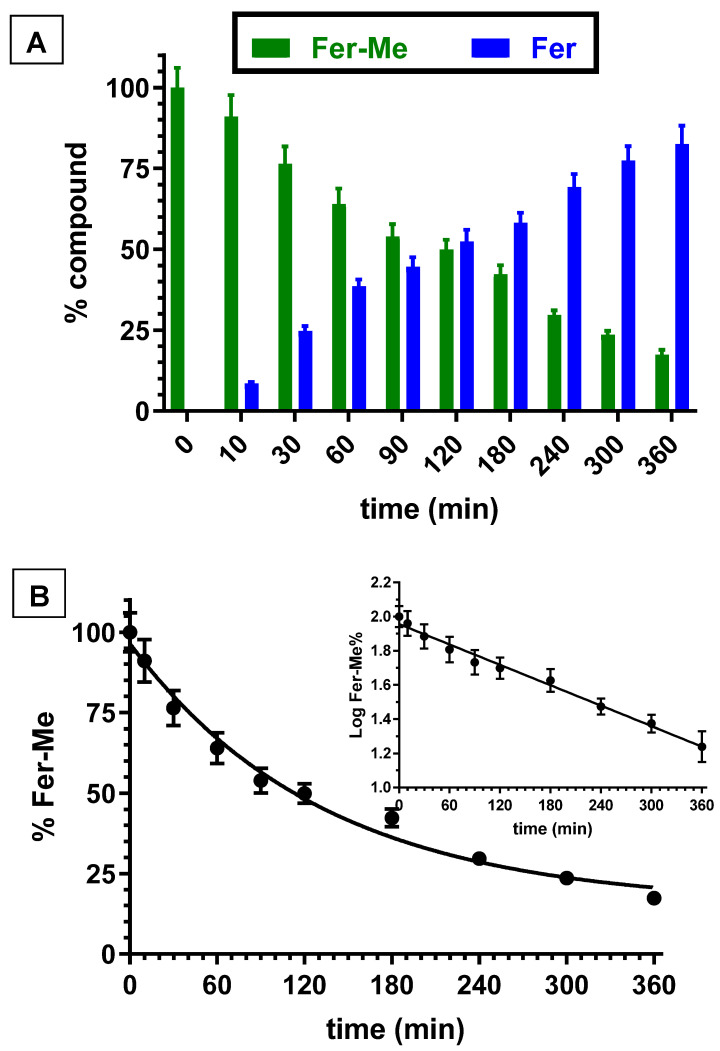
(**A**) Degradation profiles of the prodrug Fer-Me (green) and the corresponding appearance profiles of Fer (blue) in rat whole blood. All the values are reported as the percentage of the overall amount of incubated Fer-Me. Data are reported as the mean ± S.E.M. of three independent experiments. (**B**) Degradation profile of the prodrug Fer-Me in rat whole blood. Data are expressed as the mean ± S.E.M. of three independent experiments. The degradation followed a pseudo first order kinetic, confirmed by the semilogarithmic plot reported in the inset (n = 10, r = 0.994, *p* < 0.0001). The half-life of Fer-Me was calculated to be 92.4 ± 3.4 min.

The kinetic studies in whole blood or tissue homogenates were performed by adding the appropriate amounts of stock solutions in the incubation media. Fer incubated in rat whole blood or rat brain and liver homogenates was not degraded within eight hours. However, Fer-Me appeared degraded in rat whole blood and liver homogenate by a hydrolysis process. In particular, Figure 2A reports the degradation profile of Fer-Me in rat whole blood over time, evidencing a related appearance of Fer, the amounts of which increased over time. After six hours of incubation, the remaining Fer-Me was 17.3 ± 1.6% of its overall incubated amount, whereas the released Fer was 82.6 ± 5.6% of the starting Fer-Me concentration. These results indicated that Fer-Me was hydrolyzed in rat whole blood. In particular, as shown in Figure 2B, this degradation followed a pseudo first order kinetic (whose half-life—t_1/2_—was 92.4 ± 3.4 min), evidenced by the linear pattern of the semilogarithmic plot reported in the inset (n = 10, r = 0.994, *p* < 0.0001). Fer-Me could, therefore, be considered as a prodrug of Fer, being able to induce its release in rat whole blood. This behavior appears in good agreement with the well-known carboxylesterase activity in rodent plasma [37]. Accordingly, the hydrolysis of several ester prodrugs was previously identified in rat blood, together with the related release of antiviral, antitumor or antiparkinsonian agents [29,38,39].

**Figure 3:**
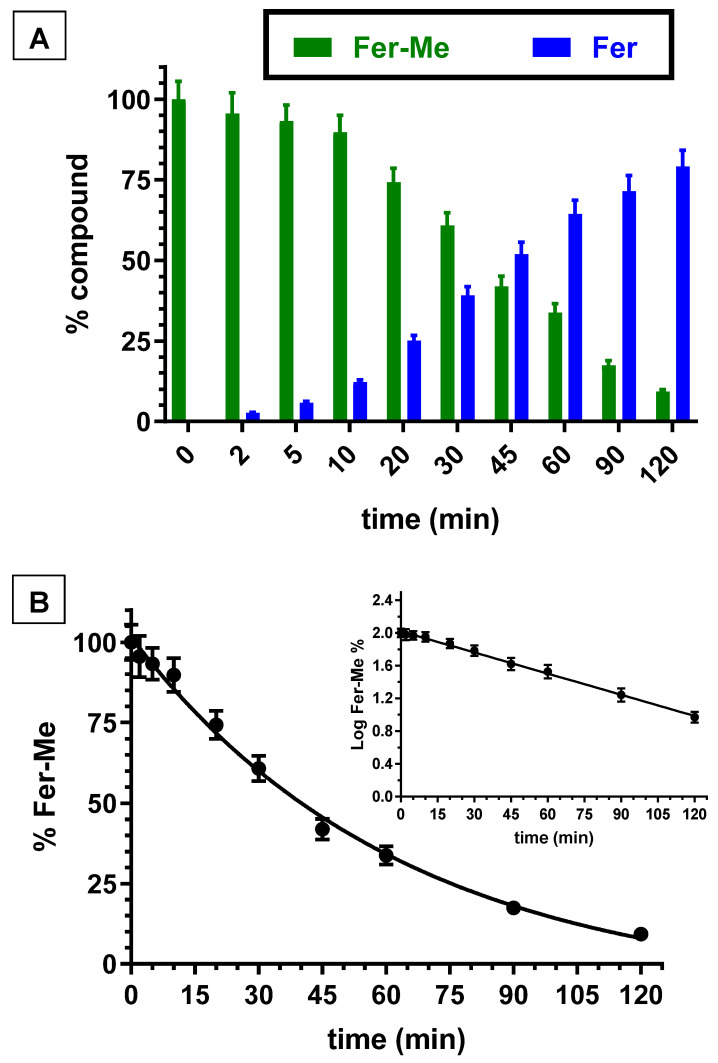
(**A**) Degradation profiles of the prodrug Fer-Me (green) and the corresponding appearance profiles of Fer (blue) in rat liver homogenate. All the values are reported as the percentage of the overall amount of incubated. Data are reported as the mean ± S.E.M. of three independent experiments. (**B**) Degradation profile of the prodrug Fer-Me in rat liver homogenate. Data are expressed as the mean ± S.E.M. of three independent experiments. The degradation followed a pseudo first order kinetic, confirmed by the semilogarithmic plot reported in the inset (n = 10, r = 0.998, *p* < 0.0001). The half-life of Fer-Me was calculated to be 34.7 ± 1.6 min.

Besides the whole blood, even rat liver homogenate was investigated for its ability to hydrolyze Fer-Me. Figure 3A reports the degradation profile of Fer-Me in rat liver homogenate over time, evidencing a related appearance of Fer, the amount of which increased over time. After two hours of incubation, the remaining Fer-Me was 9.3 ± 0.6% of its overall incubated amount, whereas the Fer that appeared was 79.2 ± 5.0% of the starting Fer-Me concentration. Fer-Me appeared, therefore, hydrolyzed in rat liver homogenate. Furthermore, in this case, as shown in Figure 3B, the degradation followed a relative fast pseudo first order kinetic (whose half-life—t_1/2_—was 34.7 ± 1.6 min), evidenced by the linear pattern of the semilogarithmic plot reported in the inset (n = 10, r = 0.998, *p* < 0.0001). These results confirmed that, at the peripheral level, Fer-Me was able to release Fer.

About the brain homogenate, Fer-Me did not evidence any hydrolysis profile, showing high stability during the six hours of incubation at 37 °C. This compound did not appear suitable to be hydrolyzed in brain homogenate, which was quite a surprising result, when it is considered that other ester prodrugs, strongly bulkier than Fer-Me, were hydrolyzed in this physiologic fluid, allowing the release of neuroactive agents [29,38]. On the other hand, this result did not preclude the chance that Fer-Me might be hydrolyzed in CNS in vivo. The antioxidant activity of this compound (very similar to that of Fer) was, anyway, here demonstrated. This aspect suggested that it might be interesting to investigate the potential anti-inflammatory activity of Fer-Me in neuronal environments, in order to verify if this compound could have any intrinsic neuroprotective activity.

### 3.4. Fer-Me Counteracts the MDP-Evoked Release of Pro-Inflammatory Cytokine TNF-α in PC12 Cells

**Figure 4:**
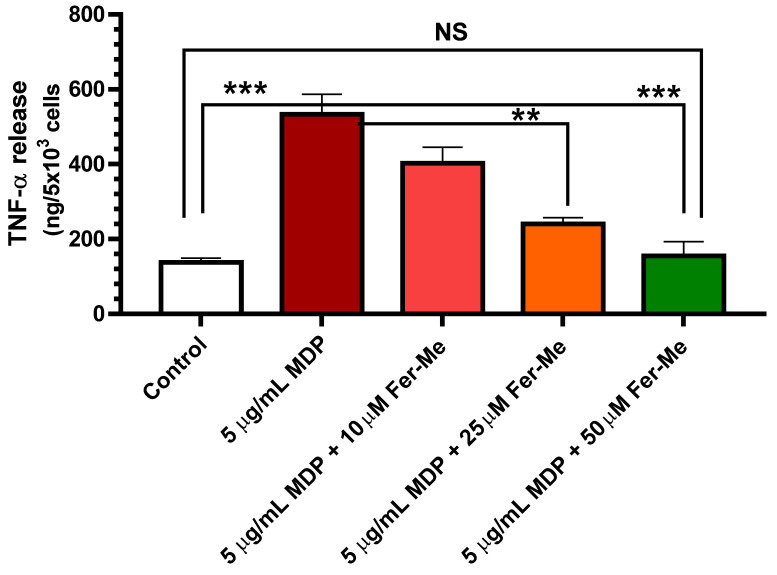
Fer-Me counteracts the release of the pro-inflammatory cytokine TNF-α evoked by 5 µg/mL MDP. PC12 cells were treated by MDP, or MDP plus increasing concentrations of Fer-Me, for 24 h. The concentrations of TNF-α in the culture supernatant were measured by ELISA. ** *p* < 0.01; *** *p* < 0.001. Results are reported as the mean ± S.E.M. of four independent experiments.

The release of pro-inflammatory cytokine TNF-α was tested to evaluate the effect of Fer-Me on MDP-evoked inflammatory injury in PC12 cells. A PC12 cell line, derived from rat pheochromocytoma, was used extensively as a model for neural differentiation, in terms of arrested proliferation and neurite outgrowth, which can be achieved by treatment with the NGF. Therefore, PC12 cells are usually suitable as an in vitro model system for neurological and neurochemical studies [40]. Here, an in vitro cell model of neuroinflammation was performed by stimulating PC12 cells with MDP. The results in Figure 4 display that release of pro-inflammatory cytokine TNF-α was significantly increased by 5 µg/mL MDP (*p* < 0.001 vs. control values). This increase was concentration-dependent (10 µM, 25 µM and 50 µM) and reduced when MDP was combined with Fer-Me, with the reduction becoming significant at 25 µM Fer-Me concentration (*p* < 0.01 vs. MDP-evoked values). Treatment with 50 µM Fer-Me alone without MDP stimulation did not significantly affect the release of TNF-α, in comparison with the control group (data not shown).

These data demonstrated that Fer-Me protects PC12 cells against MDP-evoked inflammation. In line with these findings, it is worth noting that NF-κB signaling has been reported to be up-regulated by MDP [6] and down-regulated by Fer [7], corroborating the anti-inflammatory properties of dietary polyphenols, such as Fer and its derivates, via the NF-κB signaling pathway, and being a promising therapeutic approach for several neuroinflammation arising diseases. Furthermore, it has already been shown that Fer inhibits LPS-induced TNF-α production in a dose-dependent manner in PC12 cells, expressing the maximum significant inhibitory effect at 40 μM concentration [41]. Here, MDP was preferred for testing the anti-inflammatory effects of Fer-Me, because LPS is restricted to Gram-negative bacteria, whereas muramyl peptides, such as MDP, are a widely studied signature motif of both Gram-positive and -negative species, and many immuno-compromised patients die because of gram-positive infections [42]. It is important to remark that MDP was used here for the first time in PC12 cells, confirming them as a cell model to study the neuroprotective features of candidate molecules. Our results demonstrated, therefore, that Fer-Me retained intrinsic neuroprotective activity, possibly based on its antioxidant and anti-inflammatory properties. Moreover, this compound appeared to be a candidate for encapsulation in lipidic carriers.

### 3.5. Preparation and Characterization of the SLMs

SLMs loaded with Fer or Fer-Me were obtained by a hot emulsion technique [23,26] using tristearin or stearic acid as the lipid material, being commonly used excipients in SLMs [23], and Tween 60, as a pharmaceutically acceptable emulsifier. Despite their simplicity, these microparticulate systems evidence high versatility for their formulation and purification easiness. In particular, the fusion-emulsion technique allows the microparticulate systems to be obtained in the absence of organic solvents, making the formulation methods easily reproducible in the pharmaceutical industry, and allowing sustainable practices for the environment. Moreover, the human organism shows high tolerability for these SLMs, due to the biocompatibility and biodegradability of the lipid components and the absence of organic solvent residuals.

**Figure 5:**
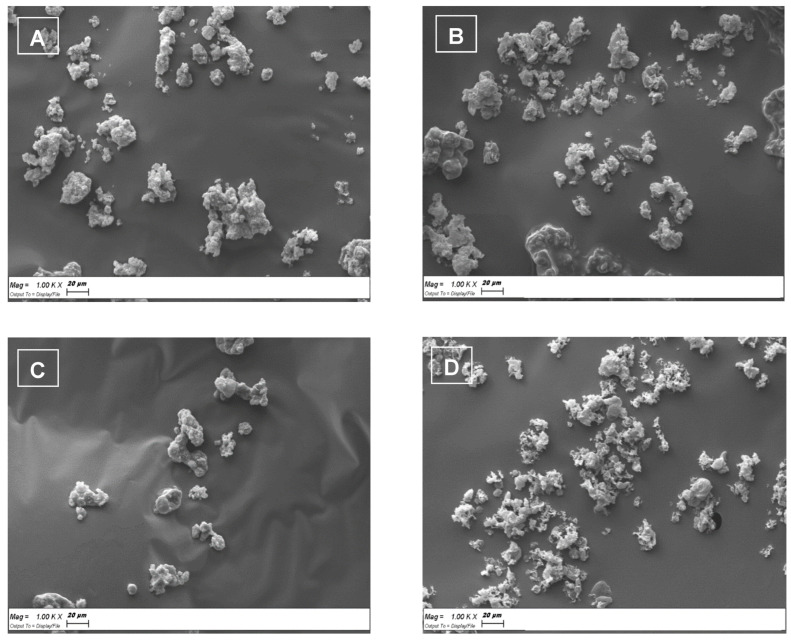
Scanning electron microscopy (SEM) micrographs of the SLMs based on tristearin or stearic acid. (**A**) Tristearin SLMs loaded with Fer; (**B**) Stearic acid SLMs loaded with Fer; (**C**) Tristearin SLMs loaded with Fer-Me; (**D**) Stearic acid SLMs loaded with Fer-Me.

Figure 5 reports the SEM micrograph of the SLMs based on tristearin or stearic acid, loaded with Fer or its derivative Fer-Me. The tristearin based SLMs loaded with Fer (Figure 5A) or Fer-Me (Figure 5C) revealed quite a spherical shape with several degrees of aggregations. The aggregates evidenced sizes ranging around 20 μm. The stearic acid based SLMs loaded with Fer (Figure 5B) or Fer-Me (Figure 5D) evidenced poorly formed particles, showing aggregates with sizes ranging, also in this case, around 20 μm.

The loading values were obtained by HPLC analysis. Chromatographic precision and calibration data are reported in the Supplementary Materials.

As reported in Table 1, the amounts of Fer encapsulated in tristearin or stearic acid based microparticles were 0.375 ± 0.004% and 0.946 ± 0.015%, respectively, which corresponded to encapsulation efficiencies of 14.9 ± 0.2% and 37.8 ± 0.6%, respectively. On the other hand, the amounts of Fer-Me encapsulated in tristearin or stearic acid based microparticles appeared greater, being 0.719 ± 0.005% and 1.507 ± 0.014%, respectively, which corresponded to encapsulation efficiencies of 27.9 ± 0.2% and 59.3 ± 0.6%, respectively (Table 1).

$$\mathrm{Drug}\;\mathrm{loading}\;(\%)=\frac{\mathrm{mass}\;\mathrm{of}\;\mathrm{drug}\;\mathrm{in}\;\mathrm{microparticles}}{\mathrm{mass}\;\mathrm{of}\;\mathrm{loaded}\;\mathrm{microparticles}}\times 100.$$

Tristearin is formed by the esterification of glycerol with three molecules of stearic acid. The alkyl chains in tristearin should have, therefore, a lower degree of freedom than in stearic acid in modifying the conformation of the lipid matrix to allow the loading of drugs. Moreover, the free carboxylic group of stearic acid should have more opportunities to exchange hydrogen bonds with the drugs, in comparison with the homologous ester groups in tristearin. These factors may contribute to increase the ability of stearic acid microparticles to load drugs, in comparison with tristearin. A similar phenomenon was observed with a prodrug of zidovudine loaded in stearic acid and tristearin microparticles [28].

**Figure 6:**
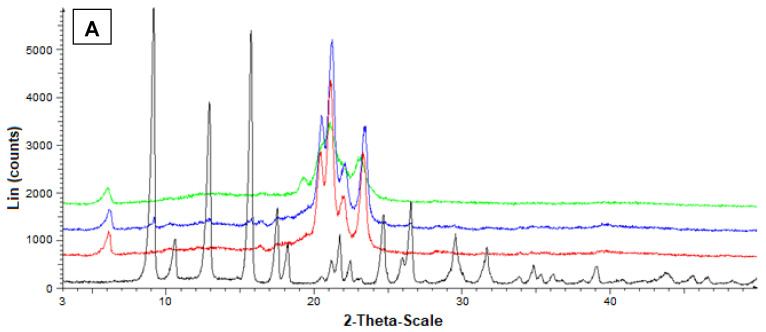

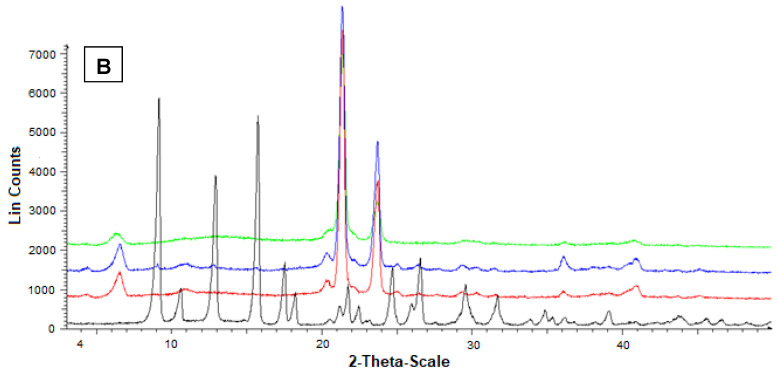
Powder X-ray diffraction patterns of Fer (black), tristearin (red) (**A**) Stearic acid (red) (**B**) Drug lipid mixtures (blue) and loaded SLMs (green). (**A**) Diffraction patterns referred to tristearin and Fer; (**B**) Diffraction patterns referred to stearic acid and Fer.

Additional information on the solid state of the SLMs was obtained by powder X-ray diffraction. In particular, Figure 6A reports the diffractograms concerning Fer (black), tristearin (red), their mixture (blue) and the related loaded SLMs (green). The black pattern of pure Fer evidenced characteristic crystalline peaks at 9°, 12° and 16°, whereas the red pattern of pure tristearin evidenced characteristic crystalline peaks at 20°, 21°, 22° and 24°. The tristearin peaks were not perturbed by the presence of mixed Fer, as evidenced by the blue pattern, where, moreover, the peaks of this drug at 9°, 12° and 16° were detectable, despite its very poor amount in the mixture with respect to the lipid. On the other hand, the green pattern referred to the loaded SLMs did not allow the detection of the peaks of Fer; moreover, the characteristic peaks of tristearin were perturbed by the loaded drug appearing spread out and without the typical definitions at 20°, 21°, 22° and 24°.

Figure 6B reports the diffractograms concerning Fer (black), stearic acid (red), their mixture (blue) and the related loaded SLMs (green). The red pattern of pure stearic acid evidenced characteristic crystalline peaks at 20.5°, 21.5°, and 24°.

Furthermore, in this case, the stearic acid peaks were not perturbed by the presence of mixed Fer, as evidenced by the blue pattern, where, moreover, the peaks of this drug at 9°, 12° and 16° were detectable, despite the very poor amount in the mixture. On the other hand, the green pattern referred to the loaded SLMs did not allow the detection of the peaks of Fer; moreover, the characteristic peaks of stearic acid were perturbed by the loaded drug appearing weakly spread out and, in particular, without the typical definition at 20.5°.

These data indicated a complete absence of interaction between drug and lipids in the mixtures, whereas Fer appeared to lose its crystallinity in loaded SLMs, where the crystalline structure of tristearin or stearic acid appeared, in turn, perturbed by the presence of the drug, suggesting, therefore, a distribution of Fer inside the lipid matrices.

**Figure 7:**
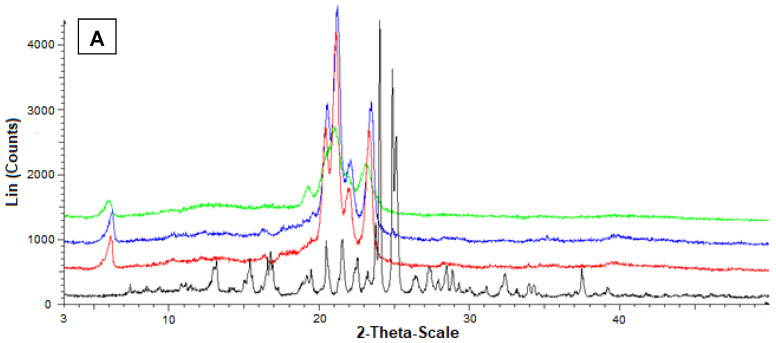

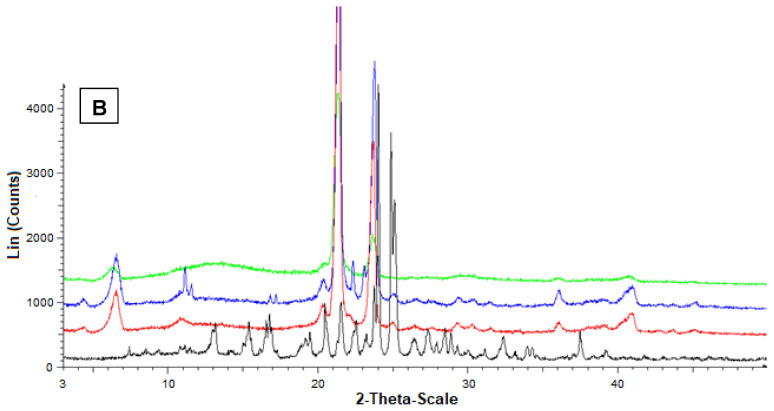
Powder X-ray diffraction patterns of Fer-Me (black), tristearin (red) (**A**) Stearic acid (red) (**B**) Drug lipid mixtures (blue) and loaded SLMs (green). (**A**) Diffraction patterns referred to tristearin and Fer-Me; (**B**) Diffraction patterns referred to stearic acid and Fer-Me.

Figure 7 reports the diffractograms obtained by the powder X-ray diffraction analysis referred to Fer-Me and tristearin (A) or stearic acid (B). In this case, the black pattern of pure Fer-Me evidenced characteristic crystalline peaks at 24° and 25°, where stearic acid evidenced very weak peaks, beyond those at 20.5°, 21.5°, and 24°. With Fer-Me, anyway, a comparison between the diffractograms of mixtures and SLMs evidenced the absence of interactions between the drug and the lipids in the mixture, whereas in SLMs the crystallinity of the lipids appeared perturbed by the presence of the drug, suggesting its distribution inside the lipid matrices.

**Figure 8:**
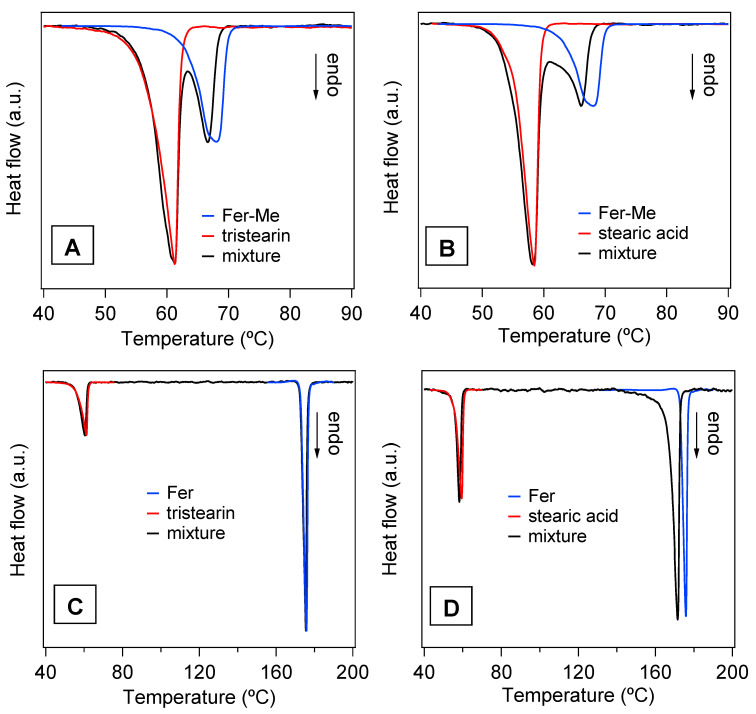
DSC curves of the four physical mixtures (black lines) and of those of the pure compounds, i.e., the lipid phases, tristearin and stearic acid (red lines) and the drug phases, Fer-Me and Fer (blue lines). (**A**) Fer-Me and tristearin; (**B**) Fer-Me and stearic acid; (**C**) Fer and tristearin; (**D**) Fer and stearic acid.

The DSC analysis performed on the mixtures of drugs with the lipids and on the SLMs provided further information about the thermal stability of these systems and the degree of interaction between their components. In particular, Figure 8 reports the DSC curves of the four physical mixtures corresponding to the different SLMs. For a direct comparison, the DSC curves of the pure compounds are also reported, i.e., of the lipid phases (tristearin and stearic acid), and of the drugs (Fer and Fer-Me). The curves of the mixtures showed endothermic peaks corresponding to those of the constituent phases and ascribed to their melting, so the solubilization of the drugs in the lipid phase could be excluded. Indeed, in the case of solubilization, just the signal of the melting of a single phase would be observed [43]. More in detail, the shape, and the melting onset (T_O_), were evaluated using the extrapolation method [44], and the peak temperature (T_P_) of the curves of the lipid phases were very close to those of the pure compounds (values of T_O_ and T_P_ for pure tristearin and stearic acid are reported in Table 2).

Differently, the melting peaks of the drugs in the physical mixtures shifted to lower temperature (Figure 8A,B,D) and/or broadened (Figure 8B,D) with respect to the pure phases. Only the Fer signal in the tristearin—Fer physical mixture appeared to be unchanged (Figure 8C).

It is known that the melting process of a sample can be affected by microstructure/size, degree of crystallinity and presence of impurities [45]. Since the investigated samples were simply obtained by mixing two pure compounds, in principle, no differences between the DSC peaks of the mixtures and of the precursors were expected. As both stearic acid and tristearin melt at lower temperatures, compared to the drugs (see Table 2), their phase transitions should not be affected by the presence of the drugs, as was indeed observed. On the other hand, during the melting process of the drugs, these were surrounded by the liquid lipid phase. The shape/position changes of the DSC peak of Fer-Me (Figure 8A,B), compared to that of the pure phase, could be due to an intermixing with the liquid lipid phase, which might develop at the beginning of the Fer-Me melting process [46,47]. Since Fer has a lower lipophilicity compared to Fer-Me [48], the same effect was not observed in the case of the tristearin—Fer mixture (Figure 8C). Regarding the stearic acid—Fer mixture, it is to be considered that the former undergoes evaporation when Fer melts [49]. The concurrence of these two processes might have affected the thermal stability of Fer and, thus, modified its peak shape/position (Figure 8D).

**Figure 9:**
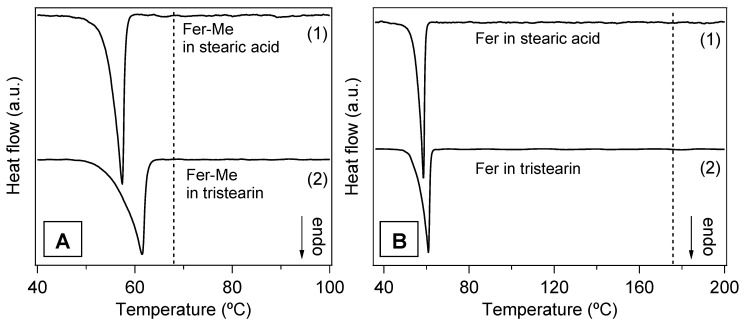
DSC curves of the SLMs. (**A**) Stearic acid SLMs loaded with Fer-Me (Fer-Me in stearic acid) (1) and tristearin SLMs loaded with Fer-Me (Fer-Me in tristearin) (2); the vertical dashed line marks the temperature T_P_ for the pure Fer-Me. (**B**) Stearic acid SLMs loaded with Fer (Fer in stearic acid (1) and tristearin SLMs loaded with Fer (Fer in tristearin) (2); the vertical dashed line indicates T_P_ of the pure Fer. The curves are displaced along the vertical axis for better visualization.

In Figure 9A,B, the DSC curves of the four samples in the form of microparticles are displayed. The endothermic peaks of the lipid phases are clearly observed, whilst no contribution ascribable to the drugs is visible. Although the amounts of drugs in the SLMs were very low, the total absence of even a faint trace of the corresponding melting peaks confirmed that the drugs were well dispersed in the lipid matrices, in line with the XRD results [21,43,50].

In Figure S3 (Supplementary Materials), the DSC signals of the lipid phases in the microparticles and in the pure form are directly compared in order to detect changes that could reveal possible modifications of the lipid microstructure. Both T_O_ and T_P_ were lower in the microparticle samples with respect to the pure compounds (see Table 2), and, particularly in the case of tristearin (Figure S3A,C), the peaks appeared broadened, especially on the left side. These features were consistent with a decrease of the melting temperature of the lipid component, due to the reduced size of the microparticles, to possible microparticles size inhomogeneity and to a decrease of their crystalline order [45,46,51,52].

These findings agree with the results of the microstructural study of the microparticles. In fact, SEM images showed a reduction of the size of the lipid phases to the order of magnitude of μm with respect to the macroscopic particles of the pure compounds and the XRD analysis clearly indicated a decrease in their crystallinity degree.

### 3.6. In Vitro Ferulic Acid or Methyl Ferulate Dissolution and Release from SLMs

The dissolution and release studies of Fer and its derivative Fer-Me were performed at 37 °C in PBS (pH 7.4), to reproduce an isosmotic fluid. The experiments were performed in order to ensure sink conditions for the studied compounds. Dissolution and release data were obtained by HPLC analysis. Chromatographic precision and calibration data are reported in the Supplementary Materials. Both Fer and Fer-Me were not degraded at 37 °C for 8 h in PBS dissolution medium.

**Figure 10:**
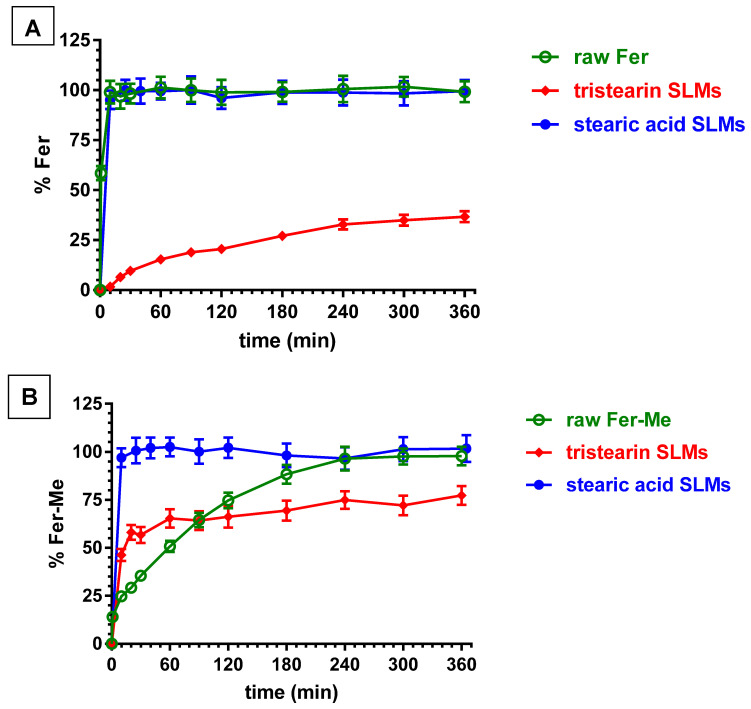
In vitro release of Fer (**A**) or Fer-Me (**B**) from SLMs based on tristearin or stearic acid. The release profiles are compared with those of the raw powder dissolution of the compounds during time. Results are reported as the mean ± S.E.M of four independent experiments.

Figure 10A illustrates the release profile of Fer from the loaded SLMs. The release patterns are compared with the dissolution of the raw powder of the drug, which showed a fast dissolution rate (about 100% within few minutes).

The tristearin based SLMs showed a release pattern characterized by a burst effect of about 6% of the incorporated Fer, followed by a relatively slow release, with less than 40% of encapsulated drug released within 6 h. These results indicated the entrapment of Fer into the lipid particle matrix, which appear able to control the release of this drug, suggesting a “core” distribution [53].

On the other hand, the SLMs based on stearic acid, despite the higher encapsulation efficiency in comparison to tristearin-based SLMs, produced a release profile superimposable with the dissolution curve of pure Fer, showing a lack of release modulation by the microparticles (about 100% within 10 min). These results indicated that Fer could be poorly localized inside the core of the microparticles, remaining absorbed into their external surface, or, alternatively, the presence of the drug in the formulative phase might induce the SLMs to produce a highly porous structure, with a consequent fast release of Fer in the dispersion medium. Differently from these results, stearic acid-based SLMs were previously evidenced to be able to encapsulate and control the release of a slightly hydrophilic anti-ischemic drug (N^6^-cyclopentiladenosine, CPA) by means of acid–base interactions between drug and lipid matrix [54]. On the other hand, the same type of SLMs were previously evidenced to be totally unable to control the release of dopamine. The synthesis of a hydrophobic prodrug of dopamine allowed the obtaining of a modulation of the release by SLMs based on stearic acid [27].

Figure 10B reports that the dissolution rate of Fer-Me in PBS was sensibly reduced in comparison to Fer. Indeed, the complete dissolution of Fer-Me required about 4 h, with respect to the few minutes related to Fer. This pattern appeared in agreement with the increased lipophilic properties of the prodrug with respect to its parent compound. It is interesting to observe that the tristearin based SLMs were able to control the release of Fer-Me, even if its release was faster with respect to the release of Fer. Indeed, the burst effect related to Fer-Me was higher than the 50% of the encapsulated prodrug, then, about the 80% of the encapsulated compound appeared released within six hours. This pattern allowed increasing of the dissolution rate of Fer-Me within the first hour of incubation in PBS, then, a moderate control of the release of the prodrug was obtained. These results suggest that Fer-Me was entrapped into the lipid particle matrix of tristearin based SLMs, even if the increased lipophilic properties of the prodrug allowed enhancement of its permeability across the lipidic phase of the particles, resulting in a faster release with respect to Fer. Moreover, the reduced size of the SLMs, as compared to the particles of raw Fer-Me, would contribute to the observed higher burst effect than dissolution rate, due to the increase in specific surface area. These phenomena appeared amplified by the SLMs based on stearic acid. Indeed, as reported in Figure 10B, the pattern of this system evidenced a complete release of the encapsulated Fer-Me within a few minutes, resulting in a strong increase of the dissolution rate of the prodrug in PBS. According to these results, Fer-Me seems, therefore, poorly localized inside the core of the microparticles, remaining absorbed into their external surface, or, alternatively, its presence in the formulative phase could induce the SLMs to produce a highly porous structure with a consequent fast release of the prodrug in the dispersion medium.

## 4. Conclusions and Future Perspectives

Fer-Me was synthesized to optimize the Fer loading in tristearin or stearic acid based SLMs. These biocompatible and biodegradable carriers can be obtained by simple formulation and purification methods, are easily scalable for industrial production and characterized by low costs and environmental impacts. Fer-Me was demonstrated as a prodrug of Fer, being hydrolyzed in physiologic fluids, such as rat whole blood and liver homogenates. Fer-Me was also characterized for its intrinsic antioxidant and anti-inflammatory properties, showing potential neuroprotective activity on neuronal-like differentiated cells. Powder X-ray diffraction studies and differential scanning calorimetry measurements evidenced the distribution of Fer-Me in the lipid matrices of SLMs, in the absence of chemical interactions.

Tristearin based SLMs were able to increase the dissolution rate of Fer-Me in water, also inducing a control of the release of both Fer and Fer-Me. Stearic acid based SLMs were able to induce a very fast dissolution of Fer-Me within a few minutes. These systems appear, therefore, to be promising carriers for the control of Fer-Me release and its targeting in the CNS. In particular, tristearin based SLMs can be proposed for intramuscular administration against neuroinflammation related to peripheral neuropathic pain. Solid lipid particulate systems are indeed known as a drug delivery platform for intramuscular and subcutaneous administration, to optimize the therapeutic effects of drugs, both at local and systemic levels [55,56,57]. Therefore, the intramuscular administration of the tristearin based SLMs loaded with Fer or Fer-Me may be proposed against neuroinflammation related to peripheral neuropathic pain, which currently requires the targeting of the neuroimmune interface for its management [5]. The potential neuroprotective effects of Fer-Me, here demonstrated by its antioxidant and anti-inflammatory activities, should be prolonged during time by the ability of tristearin SLMs to control its release. Moreover, at the peripheral level, Fer-Me can itself release Fer, contributing to a further prolonged neuroprotective effect. On the other hand, taking into account the high encapsulation efficiency and the ability to strongly promote the dissolution rate of Fer-Me, the stearic acid based SLMs appear to be a promising nasal formulation to induce the brain uptake of Fer-Me. The intranasal route can, indeed, offer an effective alternative to intravenous or oral administrations, to obtain drug or prodrug brain targeting with higher patient compliance [58]. Following nasal administration, direct permeation in cerebrospinal fluid across the olfactory mucosa is often allowed by diffusive phenomena requiring high concentrations of drugs in the nasal cavity [59]. In this regard, it has been previously demonstrated that appropriate nasal formulations can contribute to obtaining therapeutic amounts of neuroactive agents in the CNS [28,29,60,61].

## Figures and Tables

**Table 1:** Loading parameters of SLMs obtained through hot emulsion technique.

SLMs Based Lipid	Loaded Compound	Drug Loading (%)	Encapsulation Efficiency (%)
Tristearin	Fer	0.375 ± 0.004	14.9 ± 0.2
	Fer-Me	0.719 ± 0.005	27.9 ± 0.2
Stearic Acid	Fer	0.946 ± 0.015	37.8 ± 0.6
	Fer-Me	1.507 ± 0.014	59.3 ± 0.6

Data are reported as the mean ± S.E.M. of four independent experiments.

**Table 2:** Melting onset T_O_ and peak T_P_ temperature for the different pure compounds and for the lipid phase melting transition in the different formulations.

Compound	T_O_ (°C) (±0.4 °C)	T_P_ (°C) (±0.4 °C)
Stearic acid	55.7	59.5
Tristearin	56.5	61.7
Fer-Me	62.8	68.0
Fer	172.8	175.8
Stearic acid in stearic acid + Fer-Me	53.5	57.4
Stearic acid in stearic acid + Fer	54.2	58.5
Tristearin in tristearin + Fer-Me	54.9	61.5
Tristearin in tristearin + Fer	54.5	60.8

## Data Availability

Not applicable.

## Supplementary Materials

The following supporting information can be downloaded at: https://www.mdpi.com/article/10.3390/ijerph191710609/s1, S1. Synthesis of Methyl Caffeate and Methyl Ferulate; Figure S1: ^1^H-NMR spectrum of Caf-Me; Figure S2: ^1^H-NMR spectrum of Fer-Me; S2. HPLC Precision and Calibration; S3. Differential Scanning Calorimetry; Figure S3: DSC curves of the microparticle samples and of the pure lipid phases, stearic acid and tristearin.
