# Supplementary material for: Effects of Microencapsulated Ferulic Acid or Its Prodrug Methyl Ferulate on Neuroinflammation Induced by Muramyl Dipeptide
Source: Int J Environ Res Public Health. 2022 Aug 25;19(17):10609. doi: 10.3390/ijerph191710609 (PMC9518205; doi:10.3390/ijerph191710609)
Supplement: Supplementary file 1 [file ijerph-19-10609-s001.zip › ijerph-1864102-supplementary.pdf]

Supplementary Materials

Effects of microencapsulated ferulic acid or its prodrug methyl ferulate on neuroinflammation induced by muramyl dipeptide

Giada Botti, Anna Bianchi, Barbara Pavan, Paola Tedeschi, Valentina Albanese, Luca Ferraro, Federico Spizzo, Lucia Del Bianco and Alessandro Dalpiaz

S1. Synthesis of Methyl Caffeate (3) and Methyl Ferulate (4)

Methyl caffeate (Caf-Me, **3**). White powder (0.81 g; 75.4% yield). Eluent for chromatography purification: EtOAc/Petroleum ether 1:1 v/v. ^{1}H-NMR (400 MHz, DMSO-d_6, Figure S1): δ 9.34 (s, 2H), 7.46 (d, J = 15.9 Hz, 1H), 7.03 (d, J = 2.1 Hz, 1H), 6.98 (dd, J = 8.2, 2.1 Hz, 1H), 6.74 (d, J = 8.1 Hz, 1H), 6.24 (d, J = 15.9 Hz, 1H), 3.66 (s, 3H). ESI-MS: 195.25; 163.17; 236.33.

Methyl ferulate (Fer-Me, **4**). Colourless oil that became solid when cooled at 4 °C (0.82 g; 76.6% yield). Eluent for chromatography purification: EtOAc/Petroleum ether 1:2 v/v. ^{1}H NMR (400 MHz, DMSO-d_6, Figure S2): δ 9.60 (s, 1H), 7.54 (d, J = 15.9 Hz, 1H), 7.30 (d, J = 2.0 Hz, 1H), 7.10 (dd, J = 8.4, 2.0 Hz, 1H), 6.77 (d, J = 8.1 Hz, 1H), 6.45 (d, J = 15.9 Hz, 1H), 3.79 (s, 3H), 3.68 (s, 3H). ESI-MS: 209.17; 177.14; 250.17.

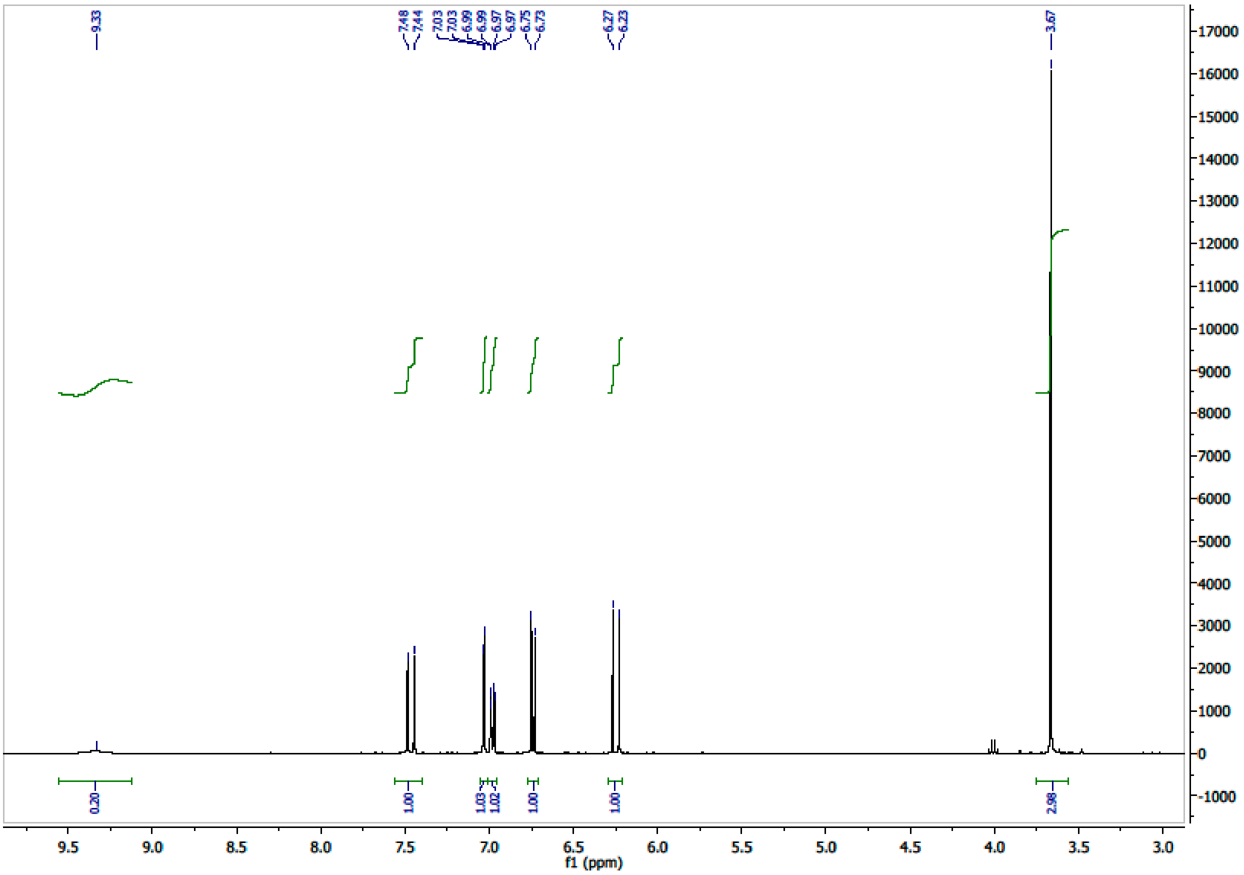

Figure S1. ^{1}H-NMR spectrum of Caf-Me.

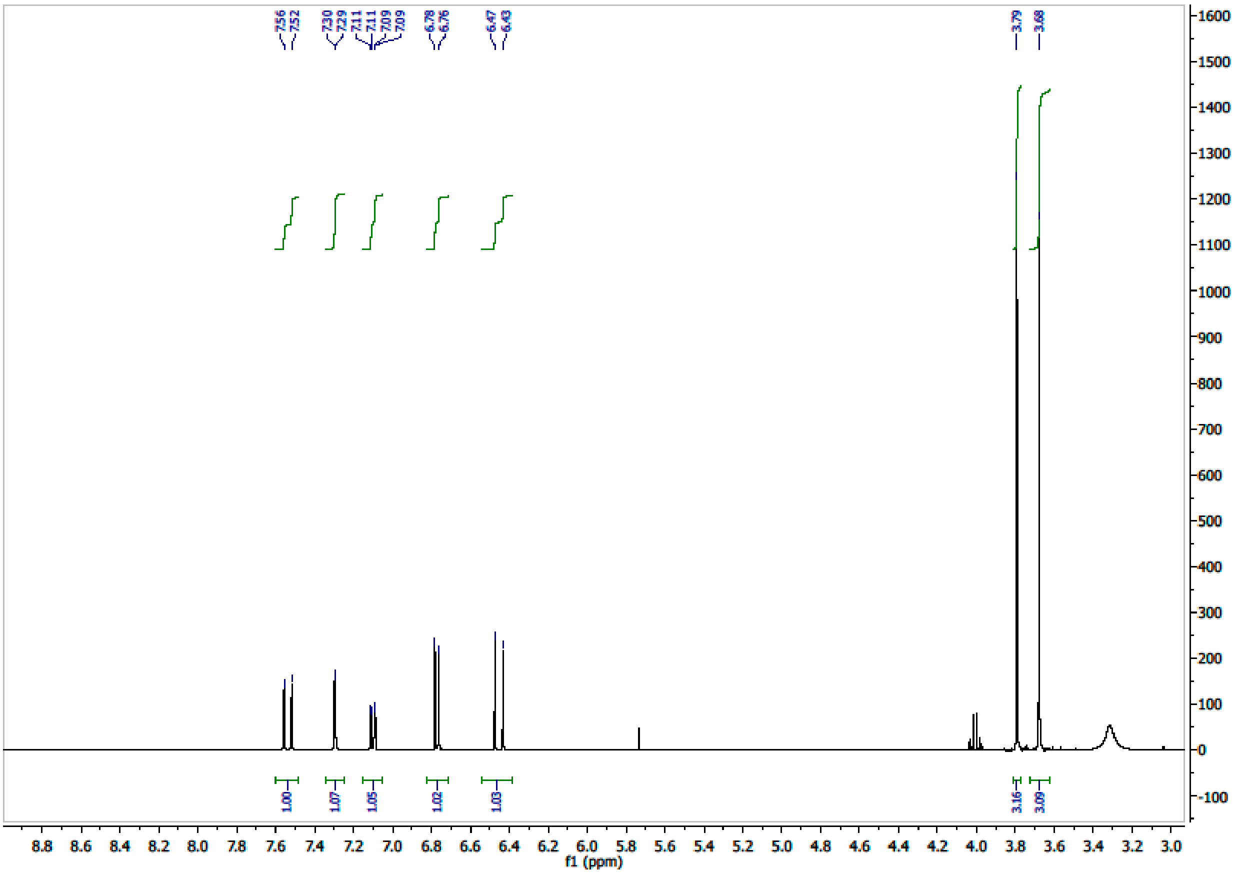

Figure S2. ^{1}H-NMR spectrum of Fer-Me.

S2. HPLC Precision and Calibration

The chromatographic precision of HPLC analytical procedures was evaluated by repeated analysis (n = 6) of the same sample solution containing each of the examined compounds dissolved in methanol, in a Tris-HCl buffer (pH 7.4), or in a phosphate buffer saline (PBS, pH 7.4) at a concentration of 50 µM. Calibration curves of peak areas *versus* concentration were generated in the range from 0.2 to 100 µM for Fer and Fer-Me.

For analysis in rat whole blood or liver and brain homogenates the accuracy of the analytical method was determined by recovery experiments, comparing the peak areas extracted from blood test samples at 4 °C (n = 6) with those obtained by the injection of an equivalent concentration of the analytes dissolved in a water and methanol mixture (50:50 v/v). For all compounds analysed, the calibration curves were constructed by using eight different concentrations in whole blood at 4 °C ranging from 0.5 to 50 µM and expressed as peak area ratios of the compounds and the internal standard *versus* concentration.

The chromatographic precision for Fer and Fer-Me dissolved in Tris-HCl buffer (the buffer where brain and liver homogenates were suspended) were represented by the relative standard deviation (RSD) values of 0.92% and 0.94%, respectively. The calibration curves of Fer and Fer-Me were linear over the range of 0.2-100 µM (n = 9, r ≥ 0.997, $P < 0.0001$). The average recoveries ± SD of the compounds from rat whole blood or rat brain and liver homogenates ranged between 42.61 ± 2.7% and 86.2 ± 3.8%. The concentrations of Fer and Fer-Me were therefore referred to as peak area ratio with respect to their internal standard Caf-Me. The precision of the method based on peak area ratio was represented by RSD values ranging between 1.3% and 1.5% for Fer and Fer-Me extracted from the different incubation media. The calibration curves referred to these compounds incubated in rat blood or rat brain and liver homogenates were linear over the range 0.5–50 µM (n = 8, r ≥ 0.988, $P < 0.0001$).

The chromatographic precision for Fer or its derivative Fer-Me dissolved in methanol was represented by RSD values of 0.95% and 0.97%, respectively. The calibration curves of each compound were linear over the range of 0.2−100 µM (n = 9, r ≥ 0.996, P < 0.0001).

The chromatographic precision for Fer and Fer-Me dissolved in PBS were represented by RSD values of 0.94% and 0.97%, respectively. The calibration curves of the compounds were linear over the range 0.2 – 100 µM (n = 9, r ≥ 0.994, P < 0.0001).

S3. *Differential Scanning Calorimetry*

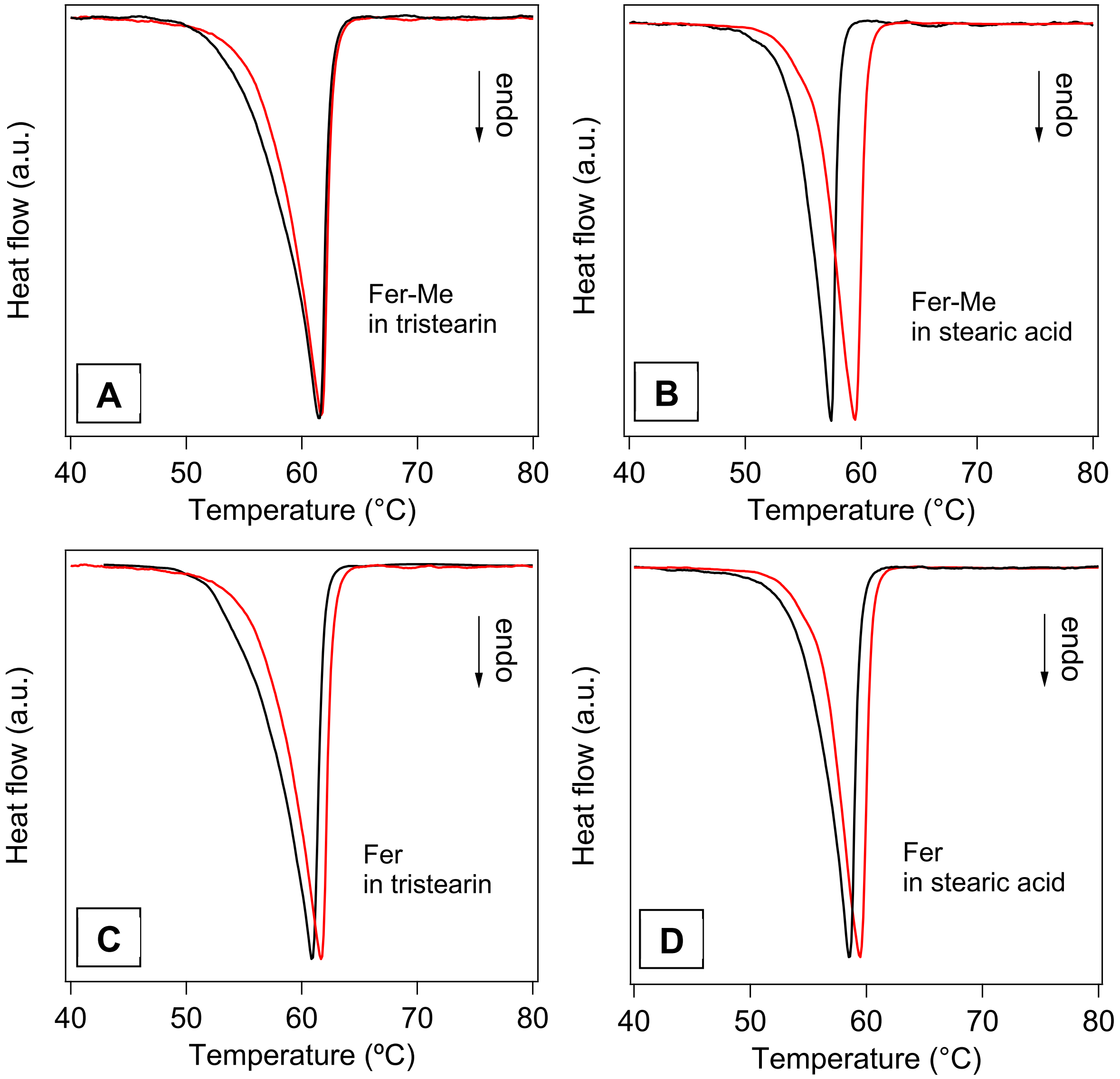

Figure S3. DSC curves of the loaded SLMs samples (black lines) and of the pure lipid phases, stearic acid and tristearin (red lines). For a better comparison, the peaks are shown as normalized to their height. [A] tristearin and Fer-Me; [B] stearic acid and Fer-Me; [C] tristearin and Fer; [D] stearic acid and Fer.
